# Are Older Adults Physically Active Enough – A Matter of Assessment Method? The Generation 100 Study

**DOI:** 10.1371/journal.pone.0167012

**Published:** 2016-11-28

**Authors:** Nils Petter Aspvik, Hallgeir Viken, Nina Zisko, Jan Erik Ingebrigtsen, Ulrik Wisløff, Dorthe Stensvold

**Affiliations:** 1 Department of Sociology and Political Science, Faculty of Social Sciences and Technology Management, Norwegian University of Science and Technology, Trondheim, Norway; 2 K.G. Jebsen Center of Exercise in Medicine at Department of Circulation and Medical Imaging, Faculty of Medicine, Norwegian University of Science and Technology, Trondheim, Norway; 3 Liaison Committee between the Central Norway Regional Health Authority and Norwegian University of Science and Technology, Trondheim, Norway; 4 School of Human Movement & Nutrition Sciences, University of Queensland, Brisbane, Australia; Universidad Europea de Madrid, SPAIN

## Abstract

**Introduction:**

Physical activity (PA) is beneficial for general health. As a result, adults around the world are recommended to undertake regular PA of either absolute or relative intensity. Traditionally, adherence to PA recommendation is assessed by accelerometers that record absolute intensity thresholds. Since ageing often results in a decrease in cardiorespiratory fitness (CRF), older adults (aged > 65 years) might be more susceptible to not meeting the PA recommendation when measured in absolute terms. The aim of the present study was to compare the adherence to the PA recommendation using both absolute and relative thresholds. Additionally, we aimed to report the reference values for overall PA in a large sample of Norwegian older adults.

**Methods:**

PA was assessed for 7 days using the Actigraph GT3X+ accelerometer in 1219 older adults (624 females) aged 70–77 years. Overall PA was measured as counts per minute (CPM) and steps. Absolute and relative moderate-to-vigorous PA (MVPA) thresholds were applied to quantify adherence to PA recommendation. The relative MVPA thresholds were developed specifically for the Generation 100 population sample. CRF was directly measured as peak oxygen uptake (VO_2peak_).

**Results:**

Proportions meeting PA recommendation were 29% and 71% when utilizing absolute and relative MVPA, respectively. More females met the relative PA recommendation compared to males. Overall PA was higher among the youngest age group. Older adults with medium- and high levels of CRF were more physically active, compared to those with the lowest levels of CRF.

**Conclusion:**

This is the first study to compare adherence to PA recommendation, using absolute and relative intensity thresholds among older adults. The present study clearly illustrates the consequences of using different methodological approaches to surveillance of PA across age, gender and CRF in a population of older adults.

## Introduction

The number of older adults (aged > 65 years of age) is expected to triple from the current 600 million to ca. 2 billion by the year 2050 [1]. An increase in life expectancy is often assumed to be accompanied by prolonged healthspan [2]. However, this is not granted, and loss of health in this population may have huge implications for the individuals quality of life and for the societal structures and economy [3]. Thus, a public health framework for action is greatly needed to maximize the healthspan of the ageing adult.

There is ample evidence that physical activity (PA) confers many health benefits even when taken up later in life [4]. With ageing, the role of PA may become even more important for independent living and for prevention of non-communicable diseases [5–7].

PA recommendation for older adults is given in both relative and absolute intensity [8, 9]. Interestingly, when adherence to PA recommendation is measured with accelerometer, a one-size-fits-all absolute intensity threshold is commonly used [10–12]. These thresholds are derived from validation studies in younger adults and may therefore not be appropriate for use in older adults [13–15]. Since ageing often results in a decrease in cardiorespiratory fitness (CRF) [16], older adults might be more susceptible to not meeting the recommendation when measured in absolute terms [13, 14].

For that reason, detailed information about overall PA and adherence to PA recommendation is vital. This will contribute to a better understanding of PA among older adults, which will be useful when planning future PA interventions in this age group [17].

The aim of the current study was to assess adherence to the current PA recommendation when PA is objectively quantified using the absolute versus relative definition. In addition, the aim was to present reference values for overall PA in terms of counts per minute (CPM), steps, and self-reported PA types, in a large sample of Norwegian older adults.

## Methods

### Study population

The present study was a cross-sectional sub-study of a larger study entitled the Generation 100 study (ntnu.edu/cerg/generation100). All males and females born between years 1936 to 1942, with a permanent address in the municipality of Trondheim were invited to participate [18]. More details regarding the Generation 100 study, including criteria for eligibility, comorbidities and risk factors of this population sample, were described elsewhere [18]. Participants with incomplete/missing accelerometer data, clinical data, and questionnaire data were excluded. In total 1219 participants, 595 (49%) males and 624 (51%) females with an age ranging from 70–77 years, were included in the present study.

The present study was approved by the Regional Committee for Medical Research (REK 2013/1903 B), and addresses baseline data from the Generation 100 study (August 2012 to June 2013). All participants gave their written informed consent, and the study was conducted in conformity with the declaration of Helsinki.

### Measures

#### Assessment of physical activity

A triaxial Actigraph GT3X+ accelerometer (Actigraph, Pensacola, Florida, USA) was used to assess PA, i.e. vertical (Y), horizontal right–left (X) and horizontal front–back axis (Z). The PA data was converted into activity counts, which reflect the intensity of bodily movement. The higher the number of counts, the more active a person is [19]. Each sample was summed over a user specified interval of time called an ‘epoch’. The epoch was set to a 10-s interval. The outcome variable is reported in uniaxial (vertical axis) and triaxial (vector magnitude) counts·min^-1^ (CPM). While uniaxial CPM has been the most common way of analyze overall PA data, triaxial CPM measures more complex movement patterns in all three planes of motion compared to uniaxial PA data [10, 20]. Step counts were calculated as a daily mean assessed by the accelerometer.

To assess adherence to PA recommendation, absolute uniaxial moderate-to-vigorous intensity PA (MVPA) (MVPA ≥ 2020 uniaxial CPM [12]) and relative triaxial MVPA (females: ≥ 1077 CPM, males: ≥ 1653 CPM [14]) were calculated. The Troiano absolute threshold applied in the present study is an established method to examine adherence to PA recommendation [11, 21, 22]. The relative intensity thresholds for MVPA were specifically developed for the Generation 100 population sample of older adults. These thresholds were developed during walking/running on a treadmill, and adjusted for gender and population CRF [14]. The triaxial CPM model was significantly better than the uniaxial CPM in predicting intensity in terms of CRF [14], and was therefore used in the current study. Both absolute- and relative MVPA were calculated by summing all minutes in sustained bouts of at least 10 min above the corresponding moderate intensity thresholds, with allowance for 2 x 1 minute interruptions below threshold. Regarding fulfillment of the national PA recommendation, the bouts of MVPA were dichotomized (meeting/not meeting), using a threshold of 150 minutes per week (from now on referred to as absolute and relative PA recommendation).

The participants received the monitor on the day they came in for clinical testing, and were instructed to wear it for 7 consecutive days. The Actilife software version 6.11.5 (Actigraph, Pensacola, Florida, USA) was used to process the accelerometer data. Non-wear time, defined as intervals of at least 60 consecutive minutes with zero counts, with allowance of 2 minutes with counts greater than zero, was excluded from the analysis [12]. All data between 6:00 a.m. and midnight were included in the analysis. Data were considered valid if the participant had at least 4 days of at least 600 min·d^-1^ recorded [12].

#### Assessment of cardiorespiratory fitness

CRF was measured as peak oxygen uptake (VO_2peak_; mL·kg^-1^·min^-1^). Briefly, testing of VO_2peak_ was performed either as walking/running on a treadmill (97%) or cycling (3%) on a stationary bike. The MetaMax II (Cortex, Leipzig, Germany) was used to test VO_2peak_. VO_2peak_ testing was initiated using inclination and speed derived from warm up and performed as an individual ramp protocol. The load was increased gradually (by either 2% inclination or 1 km/h) approximately every minute until exhaustion (VO_2peak_), or until maximal oxygen uptake (VO_2max_) was reached. Criteria used for reaching VO_2max_ were considered met when participants' oxygen uptake did not increase by more than 2 mL/kg/min in the last 60 seconds of the test (leveling off of oxygen uptake) despite increased workload and respiratory-exchange-ratio (RER) >1.05. A person’s VO_2peak_ was measured as the mean of the three successively highest 10-s VO_2_ registrations. The term VO_2peak_ was used in the current study to not exclude those who did not reach their VO_2max_ (38%). Participants with previous cardiovascular diseases were tested under ECG monitoring, and the American College of Cardiology/American Heart Association guidelines for exercise testing of patients with known cardiovascular disease were followed [23]. The output variable VO_2peak_ was continuous, and further categorized as low- (Males: <27.0 mL·kg^-1^·min^-1^, Females: <23.6 mL·kg^-1^·min^-1^), medium- (Males: 27.0–35.6 mL·kg^-1^·min^-1^, Females: 23.6–29.8 mL·kg^-1^·min^-1^) and high- (Males: >35.6 mL·kg^-1^·min^-1^, Females: >29.8 mL·kg^-1^·min^-1^) levels of CRF [14]. The distribution of participants to CRF groups is presented in Table 1.

**Table 1:** Participant characteristics.

	Male (n = 595)	Female (n = 624)
Age, yr	72.7 ± 2.2	72.9 ± 2.2
Age groups		
70–71 years	36.5	32.7
72–75 years	44.9	48.2
76–77 years	18.7	18.4
Height, cm	176.9 ± 5.6	163.4 ± 5.2
Weight, kg	82.4 ± 11.4	68.0 ± 10.6
BMI (kg·m^-2^)	26.3 ± 3.3	25.5 ± 3.7
CRF (mL·kg^-1^·min^-1^)	31.7 ± 6.8	26.3 ± 5.1
CRF groups		
Low	24.4	32.2
Medium	50.4	44.7
High	25.2	23.1

Data are presented as mean ± standard deviation or proportions (%). BMI, body mass index. Cardiorespiratory fitness (CRF) groups: Males low <27.0 mL·kg-1·min-1, Females low <23.6 mL·kg-1·min-1, Males medium 27.0–35.6 mL·kg-1·min-1, Females medium 23.6–29.8 mL·kg-1·min-1, Males high >35.6 mL·kg-1·min-1, Females high >29.8 mL·kg-1·min-1.

#### Other measures

A questionnaire was used to assess types of activities, such as walking, exercise at a fitness center, cycling, swimming etc. Frequency of activity type was measured with a 7-point scale (Never, seldom, 1–3 days per month, 1 day per week, 2–3 days per week, 4–6 days per week, daily), and dichotomized into less than once a week vs. once a week or more. Gender and age were obtained from the National Population Registry. Age was calculated from month/year of birth and month/year of inclusion, and dichotomized into 3 age groups (70–71, 72–75 and 76–77 years). Detailed protocol for assessment of body weight (kg), body height (cm) and body mass index (BMI; kg·m^-2^) has been published elsewhere [18]. Seasonal data were obtained from the activity data (month of assessment). Based on the Norwegian climate, the season variable (season) was dichotomized into “colder” (November-March) and “warmer” (April-October) months. The colder months have high probability of snow, ice and relatively few hours of daylight.

### Statistical procedures

Statistical analyses for sample characteristics, CPM- and intensity distributions were performed with PASW Statistics 21 for Windows (IBM Corporation, Somers, NY, USA). Sample characteristics are presented as means (standard deviations) and proportions (Table 1). A One-way ANOVA test (Bonferroni adjusted) and independent samples t-test were used to study the association between PA (CPM and MVPA), season, age groups, gender and CRF groups (Tables 2 and 3). Pearson chi square test was used to study associations between categorical data (proportions meeting absolute and relative PA recommendation distributed by season, age, gender and CRF) (Table 3). A P-value < 0.05 was required to declare statistical significance.

**Table 2:** Minutes of MVPA per day, and proportions meeting absolute- and relative PA recommendation.

		Absolute threshold^a^		Relative threshold^b^	
	N	MVPA^c^	Meeting PA recommendation (%)^d^	MVPA^c^	Meeting PA recommendation (%)^d^
Total	1219	16.3 (0.5)	29.0	43.8 (1.0)	71.1
Age group					
70–71 years	421	17.0 (0.9)	31.4	46.6 (2.0)	73.2
72–75 years	572	16.4 (0.7)	28.8	43.2 (1.5)	70.3
76–77 years	226	14.9 (1.1)	25.2	40.0 (1.9)	69.5
One-way ANOVA Bonferroni		P = 0.34	P = 0.26^e^	P = 0.08	P = 0.51^e^
Gender					
Male	595	17.3 (0.8)	30.3	33.7 (1.1)	62.2
Female	624	15.3 (0.7)	27.9	53.4 (1.6)	79.6
Independent-samples T test		P > 0.05	P = 0.36^e^	**P < 0.001**	**P < 0.001**^e^
CRF group					
Low	346	8.8 (0.6)^f^	14.2	29.3 (1.4) ^f^	52.3
Medium	579	16.4 (0.7)^g^	28.7	44.2 (1.5)^g^	73.7
High	294	24.9 (1.3)	47.3	60.0 (2.4)	88.1
One-way ANOVA Bonferroni		**P < 0.001**	**P < 0.001**^e^	**P < 0.001**	**P < 0.001**^e^

^a^ Absolute threshold applied to uniaxial counts per minute (Troiano et al. 2008)

^b^ Relative thresholds applied to triaxial counts per minute (Zisko et al. 2015)

^c^ MVPA, Moderate-to-vigorous PA (min·d^-1^), minutes conceded in 10 continuous minutes of MVPA per day, with allowance of 1–2 minutes drop. Mean and standard error of the mean.

^d^ 150 min/week of MVPA bouts (%), proportion of the sample who have 150 minutes of MVPA per week, conceded in 10-minute bouts.

^e^ Pearson Chi-Square test

^f^ Significantly (P < 0.05) different from the medium- and high CRF group.

^g^ Significantly (P < 0.05) different from high physical fitness group.

Cardiorespiratory fitness (CRF) groups: Males low <27.0 mL·kg-1·min-1, Females low <23.6 mL·kg-1·min-1, Males medium 27.0–35.6 mL·kg-1·min-1, Females medium 23.6–29.8 mL·kg-1·min-1, Males high >35.6 mL·kg-1·min-1, Females high >29.8 mL·kg-1·min-1.

**Table 3:** Overall physical activity, measured in CPM and steps.

	n	CPM uniaxial^a^^,^^b^	CPM triaxial^a^^,^^b^	n	STEPS^a^
Total	1219	251.2 (3.0)	502.9 (4.6)	1139	6651.9 (73.9)
Age group					
70–71 years	421	262.1 (5.5)^c^	516.7 (8.3)^c^	387	6791.6 (134.8)
72–75 years	572	247.3 (4.2)	499.6 (6.5)	526	6576.7 (109.6)
76–77 years	226	235.9 (5.7)	478.1 (9.2)	226	6587.7 (143.2)
One-way ANOVA Bonferroni		**P < 0.01**	**P < 0.05**		P = 0.40
Gender					
Male	595	253.3 (4.6)	484.5 (6.6)	564	6600.6 (108.7)
Female	624	247.4 (3.7)	517.7 (6.2)	575	6702.2 (100.5)
Independent-samples T test		P = 0.31	**P < 0.001**		P = 0.49
CRF group					
Low	346	194.4 (4.0) ^d^	419.2 (6.8) ^d^	328	5266.2 (114.0) ^d^
Medium	579	251.9 (3.9) ^e^	505.8 (6.2) ^e^	541	6762.9 (97.6) ^e^
High	294	313.0 (6.5)	590.0 (9.3)	270	8112.8 (152.8)
One-way ANOVA Bonferroni		**P < 0.001**	**P < 0.001**		**P < 0.001**

^a^ Actual means and standard error of the mean (SE) for ease of interpretation.

^b^ Uniaxial and triaxial counts per minute

^c^ Significantly (P < 0.05) different from the 76 to 77-yr group.

^d^ Significantly (P < 0.05) different from the medium- and high CRF group.

^e^ Significantly (P < 0.05) different from high physical fitness group.

Cardiorespiratory fitness (CRF) groups: Males low <27.0 mL·kg-1·min-1, Females low <23.6 mL·kg-1·min-1, Males medium 27.0–35.6 mL·kg-1·min-1, Females medium 23.6–29.8 mL·kg-1·min-1, Males high >35.6 mL·kg-1·min-1, Females high >29.8 mL·kg-1·min-1.

## Results

Participant characteristics are presented in Table 1. Of the 1219 older adults included, 59%, 33%, and 8% wore the accelerometer for 7, 6 and 4–5 valid days, respectively.

### Adherence to physical activity recommendation

Higher proportions of participants met the relative PA recommendation (71%), compared to the absolute PA recommendation (29%). Participants with high levels of CRF showed a higher adherence to both the relative- and absolute PA recommendation, compared to participants with medium and low levels of CRF (Table 2). Furthermore, more females (80%) than males (62%) met the relative PA recommendation. In contrast, the absolute PA recommendation showed no significant gender difference. The proportion of participants meeting relative- and absolute PA recommendation was similar across the age groups (Table 2). Furthermore, adherence to PA recommendation was significantly higher among participants tested in warmer months compared to those tested in colder months, for both relative (77% vs. 66%) and absolute (34% vs. 24%) MVPA thresholds. There were no seasonal differences in total registered accelerometer time. Identical associations with gender, age and level of CRF were seen when looking at MVPA bouts (Table 2).

### Overall physical activity

Participants`overall PA was positively associated with CRF, both for uniaxial and triaxial CPM, and steps (Table 3). Females had a significantly higher overall PA than males when using triaxial CPM, but not when applying uniaxial CPM. In addition, the amount of steps per day did not differ between genders. The age group 70-71yrs had a significantly higher overall PA than those aged 76-77yrs, when using both uniaxial and triaxial CPM. Amount of steps per day didn`t differ between age groups. The older adults studied in warmer vs. colder months had a significantly higher overall PA, using both uniaxial (267 vs. 235 CPM) and triaxial (524 vs. 481 CPM) data, and steps (7040 vs. 6340 steps per day).

### Physical activity types

**Fig 1:**
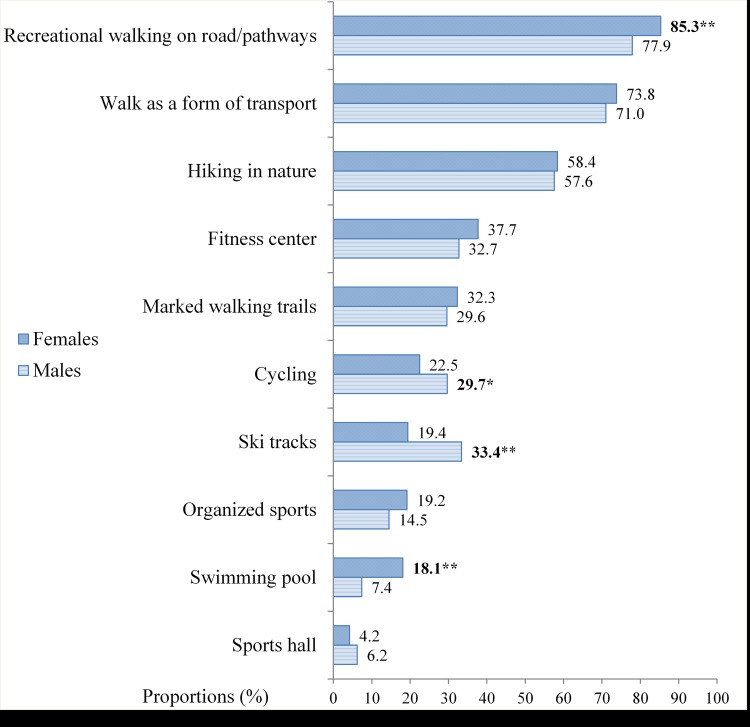
Proportions of different types of activities performed at least once a week. **Significantly (P < 0.01) different between genders, *Significantly (P < 0.05) different between genders.

Walking was the most common activity among older adults (Fig 1). Eight out of ten reported recreational walking on the road/pathways as a primary form of activity, seven out of ten reported walking as a form of transport and six out of ten reported hiking in nature at least once a week. Older adults participated in a variety of other physical activities, such as fitness center, cycling, organized sports, and swimming. More males were active on ski tracks and cycled more compared to females, while females were more active in recreational walking and swimming. There were no gender differences regarding sports, fitness center, marked walking trails, walking as a form of transport and hiking in nature.

## Discussion

The current analysis represents the first comprehensive reference data for objectively measured PA in a large sample of Norwegian older adults. The other PA reference values for comparable age groups in Norway come from a study with a much smaller sample size (n = 252) [11]. A major finding of the present study was that a much higher proportion of older adults were fulfilling the PA recommendation when PA was analyzed using relative versus absolute thresholds. Older adults with higher levels of CRF were more active than those with lower levels of CRF, both when measured as overall PA and adherence to PA recommendation. Furthermore, we found mixed results in PA across gender and age, depending on the PA outcome measures and PA data used. In addition, older adults tested in warmer months were more physically active and more likely to fulfill the PA recommendation, compared to those tested in colder months (both expressed using relative and absolute PA thresholds). Walking was the most common type of PA in this age group.

Standardized methods of surveillance (i.e. absolute MVPA threshold) have been called for to assess prevalence of PA and to allow for national and international comparisons [8, 17]. Most studies using accelerometry to assess population PA recommendation adherence, have utilized uniaxial data and absolute MVPA thresholds [10]. To compare our results to the only Norwegian study with a similar population [11], we initially applied the same criteria to our population sample. This included the use of uniaxial data, former national PA guidelines (30 min of MVPA per day) and an absolute MVPA threshold (MVPA ≥ 2020 CPM) [12]. By doing this we found similar results compared to Lohne-Seiler et al. [11], with approximately 20% of our population sample meeting the PA recommendation. In contrast, by applying the current national PA guidelines (150 min/week of MVPA) to the absolute uniaxial MVPA threshold three out of ten older adults met the PA recommendation. Our participants therefore appear to be more active than those in similar age groups of other international population studies [12, 21, 22].

The PA recommendation also acknowledges the importance of relative intensity, especially for older adults [8, 24, 25]. When applying absolute intensity thresholds older adults are more susceptible to not reaching the PA recommendation [5, 26], e.g. due to declining CRF [27, 28]. Finding a large difference in the proportions meeting absolute and relative PA recommendation, illustrates that an older adult performing relative MVPA might not reach what is commonly defined as MVPA in absolute terms, and would, as a result, be classified as not meeting the absolute PA recommendation [26]. On the contrary, when the MVPA threshold is adjusted for the population CRF and gender, that same older adult might meet the relative PA recommendation. Surveillance studies examining adherence to PA recommendation often underpin PA policies and strategies, and the choice of assessment method is therefore crucial for future focus on PA behavior among older adults.

No significant difference in the proportions of males and females meeting the absolute PA recommendation contrasts Evenson et al. [26] who found that males were more physically active than females regardless of which absolute intensity MVPA thresholds chosen. Therefore, finding females in the present study to spend more time in relative MVPA than males, contrasts both our absolute MVPA (non-significant) and the findings of Evenson et al. [26]. The use of a one-size-fits-all absolute threshold will not take into account the fact that females have a generally lower CRF compared to males [29]. It is therefore possible that older females spend more time in activities susceptible to underestimation when measured using absolute intensity MVPA than males, such as household chores [30, 31].

No significant differences between males and females when analyzing overall PA using uniaxial CPM and steps is in line with a comparable study from Norway [11]. These findings are, however, in contrast to most studies on PA among older adults, which found males to be significantly more active than females [10, 12, 26]. Interestingly, when analyzing triaxial CPM, females were found to be significantly more active than males. Since this association was not found in uniaxial CPM, it reflects the fact that triaxial CPM captures more dimensions of movement patterns (types of activities) than uniaxial CPM.

The proportion of our population sample meeting PA recommendation did not differ among age groups, neither in absolute nor in relative intensity terms. In contrast, overall PA was inversely associated with age, with significantly higher CPM (uniaxial and triaxial) in the 70-71yrs compared to the 76-77yrs category. Former studies have reported an inverse relation between age and PA assessed as both adherence to PA recommendation and overall PA. This was however found in samples with wider age spans, compared to our sample (70-77yrs) [11, 12, 32]. Since steps were equally distributed across age groups, the age group difference in CPM is likely to be explained by the inclusion of PA intensity. Our results, both across gender and age groups, clearly illustrate the consequences of choosing different PA outcome measures in surveilling PA among older adults.

CRF was included in the present study to contribute to a better description of PA. As a result, CRF was found to be positively associated with PA, measured as overall PA, time in MVPA bouts and adherence to PA recommendation. These findings are supported by previous literature [33–35]. In the present study, with a wide CRF range from 10.1–49.9 mL·kg^-1^·min^-1^ for females and 12.1–52.8 mL·kg^-1^·min^-1^ for males the relative thresholds may be prone to underestimating PA intensity in older adults with low CRF, and overestimating among those with high CRF.

The health benefits of meeting the PA recommendation in absolute versus relative intensity terms have yet to be determined, and more research is therefore needed in this regard. The intention of using relative intensity was to adjust the criteria for meeting the PA recommendation to the actual population level of fitness. Despite the fact that 70% met the relative PA recommendation, it is important to identify why the final third were not physically active enough. Such knowledge can bring us forward in regard of designing future public health strategies.

Walking, such as recreational walking, walking as a form of transport and hiking in nature, was the most common type of PA among older adults, and this is supported by earlier studies [36]. Our results also showed that older adults perform other outdoor activities such as cycling and skiing, as well as indoor activities (i.e. fitness center, sports and swimming). Our population sample of older adults measured in the warmer months were more active compared to those measured in the colder months. This could be explained by the fact that colder months (in Norway) consist of more snow, higher prevalence of ice and relatively fewer hours of daylight. Our results indicate that both outdoor environments and indoor facilities should be important focus areas for political decision makers and for future interventions to implement a physically active behavior among older adults.

### Strengths and limitations

One strength of this study is the use of an objective measure of PA in a large sample (n = 1219) of older adults. Surveillance of older adults`PA is important due to current concerns regarding the population ageing, and associated challenges [8, 17]. Most PA research has used self-reported forms of PA assessment (questionnaires) [10, 37], which are susceptible to overestimation, recall bias and social desirability, especially among older adults [10, 17, 32]. Assessing PA with accelerometers reduces many of the challenges related to self-reported measures [38]. Furthermore, the use of triaxial CPM is a relatively new way of assessing PA, and contributes to a more complex measure of PA. The accelerometer used in this study (Actigraph) records ambulatory activity well, but can underestimate activities such as cycling, skiing, resistance exercise, upper body movements and carrying loads [39]. The participants were also instructed not to use the accelerometer during aquatic activities. One out of ten of our participants reported to swim weekly. However, walking was the most frequent activity type reported among this sample of older adults, which is a type of activity that is well captured by accelerometers. This strengthens the generalizability of our results to similar age groups worldwide.

The use of a more relative assessment of PA has been called for [8, 20, 24]. Implementing relative intensity PA thresholds, specifically developed for our sample of older adults, was an attempt to develop this field of research. However, one should keep in mind that the relative thresholds don`t take into account individual CRF differences in the investigated population sample. Regarding external validity and generalizability, it is important to note that the participants were relatively healthy (i.e. self-reported health, PA and BMI) and more educated compared to the invited, but not participating, individuals [18].

## Conclusions

To date this is the largest study to combine objectively measured PA and directly measured CRF among older adults. A major finding of the present study was that PA surveillance based on the traditionally used absolute intensity could underestimate PA levels among older adults. Older adults with medium- and high levels of CRF were more physically active, compared to those with low levels of CRF. More females met the relative PA recommendation compared to males. Our results clearly illustrate the consequences of using different methodological approaches in PA surveillance.
